# {2-[(4-Bromo­phen­yl)imino­meth­yl]pyridine-κ^2^ *N*,*N*′}diiodidozinc(II)

**DOI:** 10.1107/S1600536808020680

**Published:** 2008-07-09

**Authors:** Mehdi Khalaj, Saeed Dehghanpour, Ali Mahmoudi

**Affiliations:** a Department of Chemistry, Islamic Azad University, Karaj Branch, Karaj, Iran; b Department of Chemistry, Alzahra University, Vanak, PO Box 1993891176, Tehran, Iran

## Abstract

In the title compound, [ZnI_2_(C_12_H_9_BrN_2_)], the metal centre displays a moderately distorted tetra­hedral coordination geometry defined by two iodide anions and two N atoms of the organic ligand. The dihedral angle between the pyridine and benzene rings is 15.15 (13)°.

## Related literature

For the crystal structure of similar imino­pyridine complexes, see: Dehghanpour, Mahmoudi, Khalaj & Salmanpour (2007 ▶); Dehghanpour, Mahmoudi, Khalaj, Salmanpour & Adib (2007 ▶). For related structures see: Lee *et al.* (2008 ▶); Wriedt *et al.* (2008 ▶).
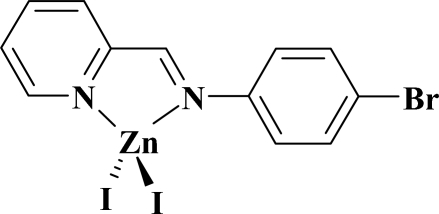


## Experimental

### 

#### Crystal data

[ZnI_2_(C_12_H_9_BrN_2_)]
*M* *_r_* = 580.29
Triclinic, 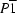

*a* = 8.0749 (9) Å
*b* = 9.7323 (11) Å
*c* = 11.1884 (13) Å
α = 79.157 (2)°
β = 71.178 (3)°
γ = 67.325 (2)°
*V* = 765.87 (15) Å^3^
*Z* = 2
Mo *K*α radiation
μ = 8.23 mm^−1^
*T* = 100 (2) K
0.45 × 0.21 × 0.12 mm

#### Data collection

Bruker APEXII CCD area-detector diffractometer
Absorption correction: multi-scan (*APEX2*; Bruker, 2005 ▶) *T*_min_ = 0.135, *T*_max_ = 0.378
9814 measured reflections
4449 independent reflections
3983 reflections with *I* > 2σ(*I*)
*R*_int_ = 0.040

#### Refinement

*R*[*F* ^2^ > 2σ(*F* ^2^)] = 0.032
*wR*(*F* ^2^) = 0.084
*S* = 1.01
4449 reflections
163 parameters
H-atom parameters constrained
Δρ_max_ = 1.94 e Å^−3^
Δρ_min_ = −1.96 e Å^−3^

### 

Data collection: *APEX2* (Bruker, 2005 ▶); cell refinement: *APEX2*; data reduction: *APEX2*; program(s) used to solve structure: *SHELXTL* (Sheldrick, 2008 ▶); program(s) used to refine structure: *SHELXTL*; molecular graphics: *SHELXTL*; software used to prepare material for publication: *SHELXTL*.

## Supplementary Material

Crystal structure: contains datablocks global, I. DOI: 10.1107/S1600536808020680/rz2225sup1.cif

Structure factors: contains datablocks I. DOI: 10.1107/S1600536808020680/rz2225Isup2.hkl

Additional supplementary materials:  crystallographic information; 3D view; checkCIF report

## Figures and Tables

**Table:** 

I1—Zn1	2.5201 (5)
I2—Zn1	2.5389 (5)
Zn1—N1	2.062 (3)
Zn1—N2	2.094 (3)

**Table:** 

N1—Zn1—N2	80.30 (11)
N1—Zn1—I1	117.81 (8)
N2—Zn1—I1	117.60 (8)
N1—Zn1—I2	109.45 (8)
N2—Zn1—I2	110.18 (8)
I1—Zn1—I2	116.210 (17)

**Fig. 1.**
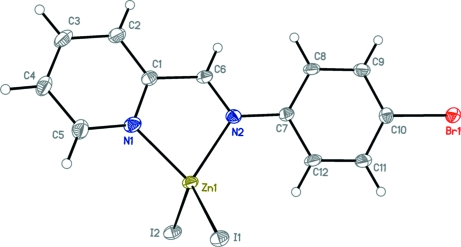
The molecular structure of the title compound showing the atom-labelling scheme and thermal ellipsoids drawn at the 50% probability level.

## supplementary crystallographic information

### Comment

Iminopyridines derivatives are common ligands and many complexes containing these ligands have been reported. Recently, the crystal structure of zinc(II)-complexes similar to the title compound has been reported by our group (Dehghanpour, Mahmoudi, Khalaj & Salmanpour, 2007; Dehghanpour, Mahmoudi, Khalaj, Salmanpour & Adib, 2007).

The molecular structure of the title compound and the atom numbering scheme are shown in Fig. 1. The structure consists of discrete [ZnI_2_(C_12_H_9_BrN_2_)] complex molecules where the metal centre has a tetrahedral coordination geometry which shows significant distortion, mainly due to the presence of the five-membered chelate ring. The endocyclic N1—Zn1—N2 angle (Table 1) is much narrower than the ideal tetrahedral angle of 109.5^°^, whereas the opposite I1—Zn1—I2 angle is much wider. Bond lengths involving the Zn atom are in good agreement with the values found in the literature for tetrahedral zinc(II) complexes (Lee *et al.*, 2008; Wriedt *et al.*, 2008). The dihedral angle formed by the pyridine and benzene ring is 15.15 (13)°. The crystal structure is enforced by van der Waals interactions only.

### Experimental

To a solution of (4-bromo-phenyl)-pyridin-2-ylmethylene-amine (26.1 mg, 0.1 mmol) in acetonitrile (20 ml) was added zinc iodide (31.9 mg, 0.1 mmol). The mixture was heated to dissolve the reactants. The solution was filtered and the volume of solvent removed under vacuum to about 5 ml. The diffusion of diethyl ether vapor into the solution gave yellow crystals. The crystals were collected and washed with diethylether-dichloromethane (9:1 *v*/*v*); yield 81%. Calc. for C_12_H_9_BrI_2_N_2_Zn: C 24.84, H 1.56, N 4.83%; found: C 24.86, H 1.55, N 4.82%.

### Refinement

All hydrogen atoms were placed geometrically and refined in isotropic approximation in riding model with the *U*_iso_(H) parameters equal to 1.2*U*_eq_(C). There is a high positive residual density of 1.93 e Å^-3^ at 0.83 Å from atom I2 due to considerable absorption effects which could not be completely corrected.

### Crystal data

**Table:**

[ZnI_2_(C_12_H_9_BrN_2_)]	*Z* = 2
*M**_r_* = 580.29	*F*_000_ = 532
Triclinic, *P*1	*D*_x_ = 2.516 Mg m^−^^3^
Hall symbol: -P 1	Mo *K*α radiation λ = 0.71073 Å
*a* = 8.0749 (9) Å	Cell parameters from 1953 reflections
*b* = 9.7323 (11) Å	θ = 2.8–34.4º
*c* = 11.1884 (13) Å	µ = 8.23 mm^−^^1^
α = 79.157 (2)º	*T* = 100 (2) K
β = 71.178 (3)º	Plate, yellow
γ = 67.325 (2)º	0.45 × 0.21 × 0.12 mm
*V* = 765.87 (15) Å^3^	

### Data collection

**Table:**

Bruker APEXII CCD area-detector diffractometer	4449 independent reflections
Radiation source: fine-focus sealed tube	3983 reflections with *I* > 2σ(*I*)
Monochromator: graphite	*R*_int_ = 0.040
*T* = 100(2) K	θ_max_ = 30.0º
ω scans	θ_min_ = 1.9º
Absorption correction: multi-scan(APEX2; Bruker, 2005)	*h* = −11→11
*T*_min_ = 0.135, *T*_max_ = 0.378	*k* = −13→13
9814 measured reflections	*l* = −15→15

### Refinement

**Table:**

Refinement on *F*^2^	Secondary atom site location: difference Fourier map
Least-squares matrix: full	Hydrogen site location: inferred from neighbouring sites
*R*[*F*^2^ > 2σ(*F*^2^)] = 0.032	H-atom parameters constrained
*wR*(*F*^2^) = 0.084	*w* = 1/[σ^2^(*F*_o_^2^) + (0.04*P*)^2^] where *P* = (*F*_o_^2^ + 2*F*_c_^2^)/3
*S* = 1.01	(Δ/σ)_max_ = 0.001
4449 reflections	Δρ_max_ = 1.94 e Å^−^^3^
163 parameters	Δρ_min_ = −1.96 e Å^−^^3^
Primary atom site location: structure-invariant direct methods	Extinction correction: none

### Special details

**Table:**

Geometry. All e.s.d.'s (except the e.s.d. in the dihedral angle between two l.s. planes) are estimated using the full covariance matrix. The cell e.s.d.'s are taken into account individually in the estimation of e.s.d.'s in distances, angles and torsion angles; correlations between e.s.d.'s in cell parameters are only used when they are defined by crystal symmetry. An approximate (isotropic) treatment of cell e.s.d.'s is used for estimating e.s.d.'s involving l.s. planes.
Refinement. Refinement of *F*^2^ against ALL reflections. The weighted *R*-factor *wR* and goodness of fit *S* are based on *F*^2^, conventional *R*-factors *R* are based on *F*, with *F* set to zero for negative *F*^2^. The threshold expression of *F*^2^ > σ(*F*^2^) is used only for calculating *R*-factors(gt) *etc*. and is not relevant to the choice of reflections for refinement. *R*-factors based on *F*^2^ are statistically about twice as large as those based on *F*, and *R*- factors based on ALL data will be even larger.

### Fractional atomic coordinates and isotropic or equivalent isotropic displacement parameters (Å^2^)

**Table:**

	*x*	*y*	*z*	*U*_iso_*/*U*_eq_	
I1	−0.28834 (3)	0.85145 (2)	0.60583 (2)	0.01640 (7)	
I2	0.16700 (3)	0.84434 (2)	0.72880 (2)	0.01874 (7)	
Br1	0.51281 (5)	0.79618 (4)	−0.04600 (3)	0.02005 (9)	
Zn1	0.02685 (5)	0.70420 (4)	0.64300 (4)	0.01456 (9)	
N1	0.0641 (4)	0.4960 (3)	0.7379 (3)	0.0178 (6)	
N2	0.2315 (4)	0.5720 (3)	0.4997 (3)	0.0154 (5)	
C1	0.2115 (5)	0.3877 (4)	0.6720 (3)	0.0159 (6)	
C2	0.2711 (5)	0.2407 (4)	0.7206 (3)	0.0195 (7)	
H2A	0.3754	0.1671	0.6722	0.023*	
C3	0.1746 (6)	0.2034 (4)	0.8421 (4)	0.0234 (7)	
H3A	0.2130	0.1039	0.8786	0.028*	
C4	0.0213 (5)	0.3136 (4)	0.9090 (4)	0.0240 (8)	
H4A	−0.0483	0.2900	0.9913	0.029*	
C5	−0.0296 (5)	0.4594 (4)	0.8543 (3)	0.0225 (7)	
H5A	−0.1335	0.5349	0.9010	0.027*	
C6	0.3009 (5)	0.4359 (4)	0.5426 (3)	0.0158 (6)	
H6A	0.4084	0.3675	0.4913	0.019*	
C7	0.3033 (4)	0.6193 (4)	0.3715 (3)	0.0147 (6)	
C8	0.4198 (5)	0.5200 (4)	0.2790 (3)	0.0169 (6)	
H8A	0.4551	0.4155	0.3005	0.020*	
C9	0.4848 (5)	0.5734 (4)	0.1550 (3)	0.0185 (7)	
H9A	0.5671	0.5061	0.0920	0.022*	
C10	0.4275 (5)	0.7263 (4)	0.1250 (3)	0.0165 (6)	
C11	0.3091 (5)	0.8267 (4)	0.2146 (3)	0.0204 (7)	
H11A	0.2710	0.9311	0.1921	0.024*	
C12	0.2468 (5)	0.7728 (4)	0.3380 (3)	0.0195 (7)	
H12A	0.1649	0.8407	0.4006	0.023*	

### Atomic displacement parameters (Å^2^)

**Table:**

	*U*^11^	*U*^22^	*U*^33^	*U*^12^	*U*^13^	*U*^23^
I1	0.01523 (11)	0.01337 (11)	0.02111 (12)	−0.00533 (8)	−0.00547 (8)	−0.00085 (8)
I2	0.01755 (12)	0.01407 (12)	0.02641 (13)	−0.00646 (9)	−0.00745 (9)	−0.00117 (9)
Br1	0.02369 (18)	0.01625 (17)	0.01622 (17)	−0.00571 (14)	−0.00288 (13)	0.00060 (13)
Zn1	0.01415 (18)	0.01008 (17)	0.01727 (19)	−0.00385 (14)	−0.00195 (14)	−0.00109 (14)
N1	0.0177 (13)	0.0139 (13)	0.0216 (14)	−0.0076 (11)	−0.0031 (11)	−0.0002 (11)
N2	0.0181 (13)	0.0132 (13)	0.0162 (13)	−0.0079 (11)	−0.0027 (10)	−0.0022 (10)
C1	0.0166 (14)	0.0140 (15)	0.0186 (15)	−0.0073 (12)	−0.0050 (12)	−0.0003 (12)
C2	0.0230 (16)	0.0138 (15)	0.0218 (16)	−0.0058 (13)	−0.0077 (13)	0.0001 (13)
C3	0.0318 (19)	0.0139 (16)	0.0265 (18)	−0.0090 (14)	−0.0136 (15)	0.0058 (14)
C4	0.0275 (19)	0.0201 (17)	0.0220 (17)	−0.0116 (15)	−0.0035 (14)	0.0056 (14)
C5	0.0230 (17)	0.0223 (18)	0.0200 (17)	−0.0092 (14)	−0.0034 (13)	0.0023 (14)
C6	0.0156 (14)	0.0111 (14)	0.0197 (16)	−0.0043 (12)	−0.0038 (12)	−0.0016 (12)
C7	0.0148 (14)	0.0123 (14)	0.0170 (15)	−0.0053 (12)	−0.0039 (11)	−0.0008 (12)
C8	0.0194 (15)	0.0097 (14)	0.0201 (16)	−0.0041 (12)	−0.0035 (12)	−0.0032 (12)
C9	0.0189 (15)	0.0138 (15)	0.0199 (16)	−0.0040 (13)	−0.0020 (12)	−0.0040 (12)
C10	0.0167 (14)	0.0178 (16)	0.0155 (15)	−0.0080 (13)	−0.0032 (12)	0.0001 (12)
C11	0.0248 (17)	0.0126 (15)	0.0206 (17)	−0.0055 (13)	−0.0031 (13)	−0.0018 (13)
C12	0.0210 (16)	0.0089 (14)	0.0213 (17)	−0.0034 (12)	0.0013 (13)	−0.0013 (12)

### Geometric parameters (Å, °)

**Table:**

I1—Zn1	2.5201 (5)	C3—H3A	0.9500
I2—Zn1	2.5389 (5)	C4—C5	1.394 (5)
Zn1—N1	2.062 (3)	C4—H4A	0.9500
Zn1—N2	2.094 (3)	C5—H5A	0.9500
Br1—C10	1.902 (3)	C6—H6A	0.9500
Zn1—N1	2.062 (3)	C7—C8	1.391 (5)
Zn1—N2	2.094 (3)	C7—C12	1.398 (5)
N1—C5	1.339 (4)	C8—C9	1.392 (5)
N1—C1	1.353 (4)	C8—H8A	0.9500
N2—C6	1.289 (4)	C9—C10	1.387 (5)
N2—C7	1.424 (4)	C9—H9A	0.9500
C1—C2	1.385 (5)	C10—C11	1.381 (5)
C1—C6	1.473 (5)	C11—C12	1.385 (5)
C2—C3	1.391 (5)	C11—H11A	0.9500
C2—H2A	0.9500	C12—H12A	0.9500
C3—C4	1.387 (6)		
			
N1—Zn1—N2	80.30 (11)	N1—C5—C4	121.8 (3)
N1—Zn1—I1	117.81 (8)	N1—C5—H5A	119.1
N2—Zn1—I1	117.60 (8)	C4—C5—H5A	119.1
N1—Zn1—I2	109.45 (8)	N2—C6—C1	119.2 (3)
N2—Zn1—I2	110.18 (8)	N2—C6—H6A	120.4
I1—Zn1—I2	116.210 (17)	C1—C6—H6A	120.4
C5—N1—C1	118.8 (3)	C8—C7—C12	119.5 (3)
C5—N1—Zn1	128.7 (3)	C8—C7—N2	123.0 (3)
C1—N1—Zn1	112.4 (2)	C12—C7—N2	117.5 (3)
C6—N2—C7	120.8 (3)	C7—C8—C9	120.2 (3)
C6—N2—Zn1	111.5 (2)	C7—C8—H8A	119.9
C7—N2—Zn1	127.5 (2)	C9—C8—H8A	119.9
N1—C1—C2	122.7 (3)	C10—C9—C8	118.9 (3)
N1—C1—C6	115.2 (3)	C10—C9—H9A	120.5
C2—C1—C6	122.1 (3)	C8—C9—H9A	120.5
C1—C2—C3	118.4 (3)	C11—C10—C9	121.8 (3)
C1—C2—H2A	120.8	C11—C10—Br1	120.0 (3)
C3—C2—H2A	120.8	C9—C10—Br1	118.1 (3)
C4—C3—C2	119.0 (3)	C10—C11—C12	118.9 (3)
C4—C3—H3A	120.5	C10—C11—H11A	120.6
C2—C3—H3A	120.5	C12—C11—H11A	120.6
C3—C4—C5	119.3 (3)	C11—C12—C7	120.6 (3)
C3—C4—H4A	120.3	C11—C12—H12A	119.7
C5—C4—H4A	120.3	C7—C12—H12A	119.7
			
N2—Zn1—N1—C5	−175.2 (3)	Zn1—N1—C5—C4	−175.4 (3)
I1—Zn1—N1—C5	−59.0 (3)	C3—C4—C5—N1	0.9 (6)
I2—Zn1—N1—C5	76.7 (3)	C7—N2—C6—C1	−174.2 (3)
N2—Zn1—N1—C1	9.0 (2)	Zn1—N2—C6—C1	10.1 (4)
I1—Zn1—N1—C1	125.1 (2)	N1—C1—C6—N2	−2.6 (5)
I2—Zn1—N1—C1	−99.1 (2)	C2—C1—C6—N2	175.4 (3)
N1—Zn1—N2—C6	−10.3 (2)	C6—N2—C7—C8	14.4 (5)
I1—Zn1—N2—C6	−126.7 (2)	Zn1—N2—C7—C8	−170.5 (3)
I2—Zn1—N2—C6	97.0 (2)	C6—N2—C7—C12	−168.0 (3)
N1—Zn1—N2—C7	174.3 (3)	Zn1—N2—C7—C12	7.0 (4)
I1—Zn1—N2—C7	57.9 (3)	C12—C7—C8—C9	2.2 (5)
I2—Zn1—N2—C7	−78.4 (3)	N2—C7—C8—C9	179.7 (3)
C5—N1—C1—C2	−0.8 (5)	C7—C8—C9—C10	−1.7 (5)
Zn1—N1—C1—C2	175.5 (3)	C8—C9—C10—C11	0.5 (5)
C5—N1—C1—C6	177.2 (3)	C8—C9—C10—Br1	−178.3 (3)
Zn1—N1—C1—C6	−6.5 (4)	C9—C10—C11—C12	0.3 (6)
N1—C1—C2—C3	0.2 (5)	Br1—C10—C11—C12	179.0 (3)
C6—C1—C2—C3	−177.6 (3)	C10—C11—C12—C7	0.2 (6)
C1—C2—C3—C4	0.9 (6)	C8—C7—C12—C11	−1.5 (5)
C2—C3—C4—C5	−1.5 (6)	N2—C7—C12—C11	−179.1 (3)
C1—N1—C5—C4	0.2 (5)		
